# Characterization of m6A regulator‐mediated methylation modification patterns and tumor microenvironment infiltration in acute myeloid leukemia

**DOI:** 10.1002/cam4.4531

**Published:** 2022-01-13

**Authors:** Shiyu Han, Jiaqian Qi, Kun Fang, Hong Wang, Yaqiong Tang, Depei Wu, Yue Han

**Affiliations:** ^1^ National Clinical Research Center for Hematologic Diseases Jiangsu Institute of Hematology The First Affiliated Hospital of Soochow University Suzhou China; ^2^ Institute of Blood and Marrow Transplantation Collaborative Innovation Center of Hematology Soochow University Suzhou China; ^3^ Institute of Blood and Marrow Transplantation Suzhou China; ^4^ Key Laboratory of Thrombosis and Hemostasis of Ministry of Health Suzhou China; ^5^ University of Washington Seattle Washington USA; ^6^ State Key Laboratory of Radiation Medicine and Protection Soochow University Suzhou China

**Keywords:** immunotherapy, leukemia, m6A, microenvironment, mutation burden

## Abstract

**Background:**

Previous studies have confirmed the existence of epigenetic regulation of immune responses in acute myeloid leukemia. However, the potential role of RNA N6‐methyladenosine (m6A) remodeling in tumor microenvironment (TME) infiltration remains unclear.

**Methods and Materials:**

m6A patterns of 469 AML patients (420 of which provided survival data) based on 18 m6A regulators were systematically evaluated. Based on the expression of 18 m6A regulators, unsupervised agglomerative cluster analysis was applied to recognize the various m6A modification types and to classify patients. We linked these patterns to TME infiltration characteristics and identified three distinct populations of m6A modifications.

**Results:**

These three TME cell infiltration patterns are characterized by a high degree of concordance with the three tumor immunophenotypes, which include immunoinflammatory, immunorejection, and immune inert patterns. We showed that assessment of m6A modification patterns within individually neoplasms can forecast the stage of neoplasmic inflammation, TME basal activity, subtype, hereditary mutation, and clinical patient prognosis. Limited low m6Ascore, featuring increased mutational load and immune activation, indicates an inflammatory phenotype of TME with a 5‐year survival rate at 14.4% compared to the high‐m6Ascore group (40.9%).

**Conclusions:**

Data from two different cohorts demonstrated that a higher m6Ascore showed a marked therapeutic superiority as well as clinical advantage. Assessing m6A modification patterns in AML patients could improve our knowledge of the TME infiltrative profile as well as directing effective immunotherapeutic approaches.

## Graphical abstract

TME cell infiltration patterns are characterized by a high degree of concordance with the three tumor immunophenotypes, which include immunoinflammatory, immune rejection, and immune inert patterns. Assessment of m6A revision patterns within individual neoplasms can forecast the stage of neoplasmic inflammation, TME basal activity, subtype, hereditary mutation, and clinical patient prognosis. Limited low m6Ascore, featuring increased mutational load, and immune activation, indicates an inflammatory phenotype of TME with a 5‐year survival rate at 14.4% compared to the high‐m6Ascore group (40.9%).

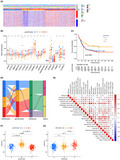

## 1 INTRODUCTION

In organisms, over 150 RNA types of modifications were identified as epigenetic layer III, including N1‐methyladenosine (m1A), N6‐methyladenosine (m6A), and 5‐methylcytosine (m5C).^1^ Of these alterations, m6A RNA methylation, broadly present in lncRNA, mRNA, and miRNA, is the most outstanding as well as essential status of intrinsic modification in Eukaryotic cells, comprising around 0.4% of the overall adenosine resident abundance.^2^, ^3^, ^4^ Analogous to the pattern of protein as well as DNA modifications, m6A modification is a variable and reproducible procedure in mammalian cells, modulated by binding proteins, demethylases, and methyltransferases (also regarded as “readers,” “writers,” and “erasers”).^5^ M6A methylation is regulated by METTL3, ZC3H13, RBM15, METTL14, KIAA1429, as well as WTAP to perform the methyltransferase, and the removal procedure is performed by demethylases, including FTO and ALKBH5. Furthermore, specific RNA binding proteins consisting of HNRNPA2B1, YTHDC1/2, YTHDF1/2/3, FMR1, and LRPPRC can recognize the m6A pattern and affect m6A function.^6^, ^7^ An in‐depth study of those regulators will help to uncover the functions and mechanisms of m6A modification in the posttranscriptional modulation. The m6A regulators have been reported to have an important role in various biological features in vivo.^8^, ^9^ A great number of evidence suggests that dysregulation of m6A regulators expression and genetic changes are associated with dysregulation of multiple procedures, including developmental defects, dysregulation of cell proliferation, abnormal immune regulation, impaired self‐renewal capacity, and neoplasms progression.^10^, ^11^

Traditionally, tumor development has been considered a step‐by‐step procedure which involves only the epigenetic and genetic variation of neoplastic cells. However, several investigations have demonstrated that the microenvironment in that neoplastic cells develop and exist also has a great impact on the progression of tumors. The neoplasm component consists of a complex tumor microenvironment (TME) containing cancer cells and stromal cells.^12^

In acute myeloid leukemia (AML), m6A within mRNA is the predominant substrate of FTO. FTO performs an oncogenic function in AML cells by promoting proliferation and inhibiting differentiation. FTO is widely expressed on the cytoplasm and has shown to drastically demethylate cytoplasmic m6A in mRNA, connecting the function of FTO in oncogenesis to its cytoplasmic m6A demethylation activity.^13^ Vu et al. also showed that METTL3 in AML is expressed at high levels and exerts a crucial role in the survival and leukemia progression of AML cells by facilitating the translation of mRNAs, including BCL2, MYC, and PTEN, in an m6A‐dependent way.^14^ WTAP has been proven to be highly upregulated in AML and to function as an oncogene. It was subsequently identified as a constituent of the m6A methyltransferase complex.^15^

In contrast, antitumor effects are characterized by many tumor suppressors interacting in a relative way. Thus, a thorough overview of the TME infiltration characteristics conducted by various m6A modulators could help enhance the recognition of TME immune modulation. At present, we consolidated genetic data from 469 AML samples to fully assess m6A modification profiles and correlated m6A modification modes with TME cell infiltration patterns. We uncovered three different m6A modification patterns and were surprised to find that TME profiles in those three patterns were strongly aligned with inflammation, immune rejection, and immunodeficiency phenotype, respectively. This suggests that m6A modifications play a significant role in molding the characteristics of personal neoplasms microenvironment. For this reason, we developed a rating system to identify the pattern of m6A modification in every patient.

## 2 METHODS AND MATERIALS

### 2.1 Acute myeloid leukemia dataset sources and preprocessing

In this study, we obtained public data with genetic expression and full clinical annotation from The Cancer Genome Atlas (TCGA) and Gene Expression Omnibus (GEO) databases. Patients who did not have survival data were excluded from further assessment. A total of eligible GC cohorts (GSE23312 and TCGA‐LAML) were captured for evaluation. We downloaded the raw files and used the strong multi‐array averaging approach of the “simpleaffy” and “affy” packages for context modification and quantitative normalization (Figure S1). The RNA‐seq data were analyzed by PCA (Figure S2). We directly downloaded the normalized matrix files in platform. For the TCGA dataset, gene expression RNA‐seq data (FPKM values) were obtained from the Genomic Data Commons (https://portal.gdc.cancer.gov/) via TCGA‐biolinks^16^ with R package, specifically developed for the integration of GDC data analysis developed.^16^ FPKM values were then converted to transcriptome per kilobase values. The “ComBat” algorithm of the “sva” package was applied to correcting for batch‐effects from non‐biotechnology bias. Data of somatic mutation were obtained from the TCGA. Data were analyzed with R (v4.0.2).

### 2.2 Clustering analysis of 18 m6A regulators

We extracted a total of 18 regulators from the GEO and TCGA datasets to recognized different m6A modifications. These 18 m6A regulators contains two erasers (FTO, ALKBH5), four writers (ZC3H13, WTAP, RMB15B, RMB15), and 12 readers (YTHDC2, YTHDC1, YTHDF2, YTHDF1, HNRNPC, HNRNPA2B1, LRPPRC, FMR1, IGBP1, IGBP2, IGBP3, RBMX). On the basis of the expression of 18 m6A regulators, unsupervised agglomerative cluster analysis was applied to recognize the various m6A modification types and to classify patients. A classification algorithm determined the clusters and their stability. The “ConsensuClusterPlus” package followed the above steps and performed 1000 iterations to ensure the strength of the classification.^17^

### 2.3 Functional annotation and genomic variation analysis (GSVA)

To examine differences in bioprocesses among m6A modification patterns, we conducted GSVA enrichment with the “GSVA” package. It is a unsupervised and nonparametric way to estimate pathways and variations in bioprocess activity of samples in an expert dataset.^18^ The set “c2.cp.kegg.v6.2” was obtained from the MSigDB library by loading the symbols and used to run analysis. A *p*‐values < 0.05 were regarded as being statistically significant. Functional annotation of m6A‐related genes was carried out with the “clusterProfiler” R package with a cutoff of FDR < 0.05.

### 2.4 Evaluation of cellular infiltration in TME

We applied the ssGSEA method to assess each infiltration in TME. The set identifying each TME immune type was derived from Charoentong's investigation. It stores various immune subtypes contains activated dendritic cells, activated CD8 T cells, regulatory T cells, macrophages, and natural killer T cells.^19^, ^20^ The relative abundance of TME‐infiltrated cells in the samples was quantified using the enrichment fraction of ssGSEA.

### 2.5 Differentially expressed genes (DEGs) between different phenotypes of m6A

To evaluate the genes associated with m6A, we calculated the expression of 18 m6A regulators, classifying patients into three different m6A modification patterns. The “limma” package was used to identify the DEGs between the different modification patterns. Critical region for identity the DEGs was set to a modified *p* < 0.01.

### 2.6 Evaluation of the m6A gene signature

To identify the m6A modification patterns of tumors, we established a rating system to assess the m6A modification patterns of AML patients. The procedure to establish the m6A gene signature is as follows. DEGs in all AML samples identified from different m6A clusters were first normalized, and then overlapping genes were extracted. Gene‐cluster is a different gene set obtained by clustering the genes involved in the patient's RNA‐seq according to different correlations and expression changes based on clustering analysis.

DEGs identified from different m6A clusters were first normalized in all samples and overlapping genes were extracted. The overlapping DEGs were analyzed by using an unsupervised clustering method to divide the patients into groups for more in‐depth analysis. Consensus clustering algorithms were used to define the number of gene clusters as well as their stability. We then performed a prognostic analysis for each gene in the signature using a univariate Cox regression model. Genes with significant prognosis were extracted for further analysis. Then, we performed principal component analysis (PCA) to construct the m6A‐associated gene signatures. Both principal components 1 and 2 were selected as signature scores. This approach has the advantage of focusing the scores on the set with the largest block of related (or anti‐related) genes, while reducing the contribution of genes that are not related to other set members. We then define the m6Ascore in a similar way to the GGI.^21^, ^22^

m6Ascore=Sum(PC1i+PC2i)

where i is the expressed gene of the m6A phenotype‐related gene.

### 2.7 Correlation of m6A gene features with other biological procedure

Mariathasan et al. built a cluster storing genes related to several biological processes, comprising (1) Antigen dealing mechanisms built a gene set that stores genes related to several biological processes, including (2) Immunity checkpoints, (3) epithelial‐mesenchymal transition (EMT) markers, (4) CD8 T‐effector signature, (5) pan‐fibroblast TGF‐β response signature, (6) angiogenic signature, (7) targets of WNT, (8) mismatch repair, (9) repair of DNA damage, (10) nucleotide excision repair, (11) antigen processing and presentation, (12) DNA replication.^23^, ^24^ We also conducted correlation analyses to uncover links between m6A gene signatures and biological pathways.

### 2.8 Statistical analysis

Correlation coefficients between m6A regulator and TME‐infiltrating immune cell expression were obtained by Spearman and distance correlation analysis. Kruskal–Wallis test and One‐way ANOVA were applied to compare the variation between three or more groups.^25^ Using the “survminer” R package, cut points for each data set by subgroup were analyzed based on the link between patient survival and m6Ascore. The “surv‐cutpoint” package was applied to retrieve all potential cut points to find the maximum grade to slice the m6Ascore. We set high and low groups based on the maximum grade to suppress the calculated batch‐effect. Multivariate Cox regression models determined independent prognostic variables. M6Ascore sensitivity and specificity were evaluated by receiver operating characteristic curves and the area under the curve was analyzed via the “pROC” package. “maftools” package was applied to illustrate mutations in patients with high and low‐m6Ascore subtypes in the TCGA‐AML cohort. “RCircos” was applied to map the variation of m6A regulators in chromosome pairs.^26^ *p*‐values were bilateral, and *p* < 0.05 was considered statistically significant.

## 3 RESULTS

### 3.1 Expression and genetic changes of m6A regulators in acute myeloid leukemia

**FIGURE 1:**
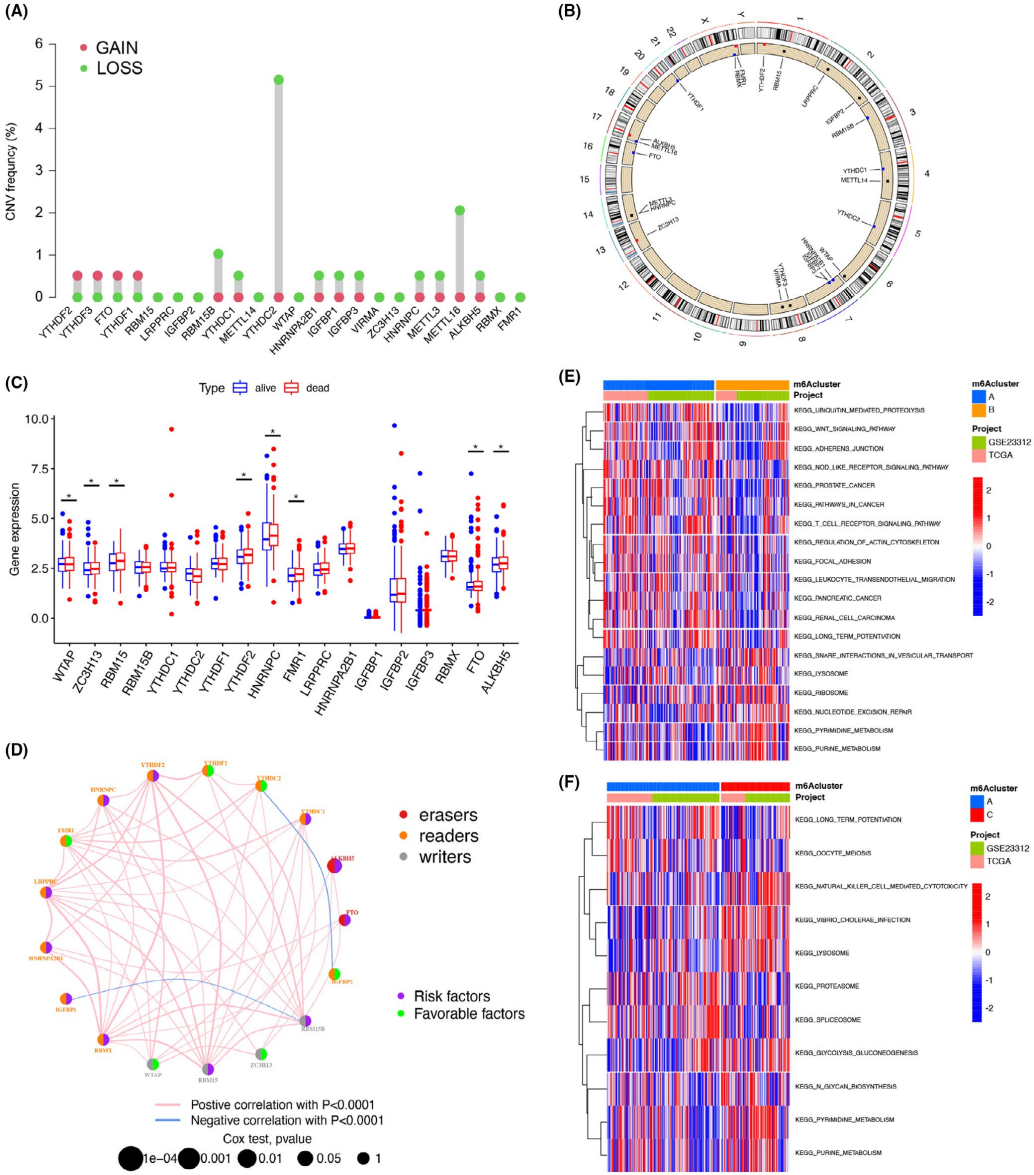
Expression and genetic variation of m6A regulators in acute myeloid leukemia. (A) CNV variation frequencies of m6A regulators in the TCGA queue. The height of the columns represents the frequency of alteration. Green dots are deletion frequencies; red dots are amplification frequencies. (B) The location of CNV changes in the m6A regulator on 23 chromosomes was studied using the TCGA cohort. (C) Expression of 18 m6A regulators in the GEO (GSE23312) and TCGA databases, with survival data for a total of 420 samples. Survival, blue; Death, red. The lower and upper ends of the boxes represent the interquartile range of values. The lines in the boxes indicate the median and the dots indicate outliers. (D) Interactions between m6A regulators in AML. The circle size indicates the effect of each moderator on prognosis, and the range of values calculated by log‐rank test is *p* < 0.001 to *p* < 0.1. Green dots in the circles, prognostic risk factors; black dots in the circles, prognostic protective factors. The line connecting the moderators indicates their interaction, and the thickness indicates the strength of the correlation between the moderators. Negative correlations are marked in blue, and positive correlations are marked in red. Regulator groups A–C are marked in red, yellow, and gray, respectively. GSVA enrichment analysis shows the activation status of biological pathways under different m6A modification patterns. Heat maps were used to visualize these biological processes. Red represents activated pathways and blue represents repressed pathways. (E) m6Acluster‐A vs. m6Acluster‐B; (F) m6Acluster‐A vs. m6Acluster‐C

Eighteen m6A regulators were identified, comprising two erasers, four writers, and 12 readers. Investigation of the frequency of CNV alterations revealed that CNV alterations was prevalent in 14 of the 18 regulators. Most of which were focused on the loss of copy number. At the same time, YTHDC2 had a wide range of CNV deletion frequencies (Figure 1A). The location of the CNV changes in the m6A regulator on the chromosome is shown in Figure 1B. To determine whether genetic variants affect the regulation of m6A expression and its prognosis in AML patients, we surveyed the mRNA levels of the regulator between “alive” and “dead” specimens of AML patients and discovered that changes in CNV may be a distinguishing factor in the disruption of m6A regulator expression. Expression of CNV‐amplified m6A regulators was elevated among the AML with poor prognosis compared to the good prognosis group (Figure 1C). This analysis above indicates a high degree of heterogeneity in the genetic and expression alterations of m6A regulators in AML samples with different prognosis, suggesting that imbalance in m6A regulator expression plays an important role in the progression of AML.

### 3.2 m6A methylation modifications controlled by 18 regulators

TCGAs and the GEO dataset (GSE23312) with available clinical information and OS data were included in a meta‐queue. We used an m6A regulator network to describe the interactions of m6A regulators in AML samples, regulators' association, and their prognostic significance for AML patients (Figure 1D). We discovered a clear correlation in the m6A regulator expression within the same functional class and between writers, erasers, and readers. The above results suggest that the interconversion between regulators of readers, writers, and erasers may affect on forming different m6A modification patterns.

### 3.3 Characterization of TME cell infiltration under different m6A modification

**FIGURE 2:**
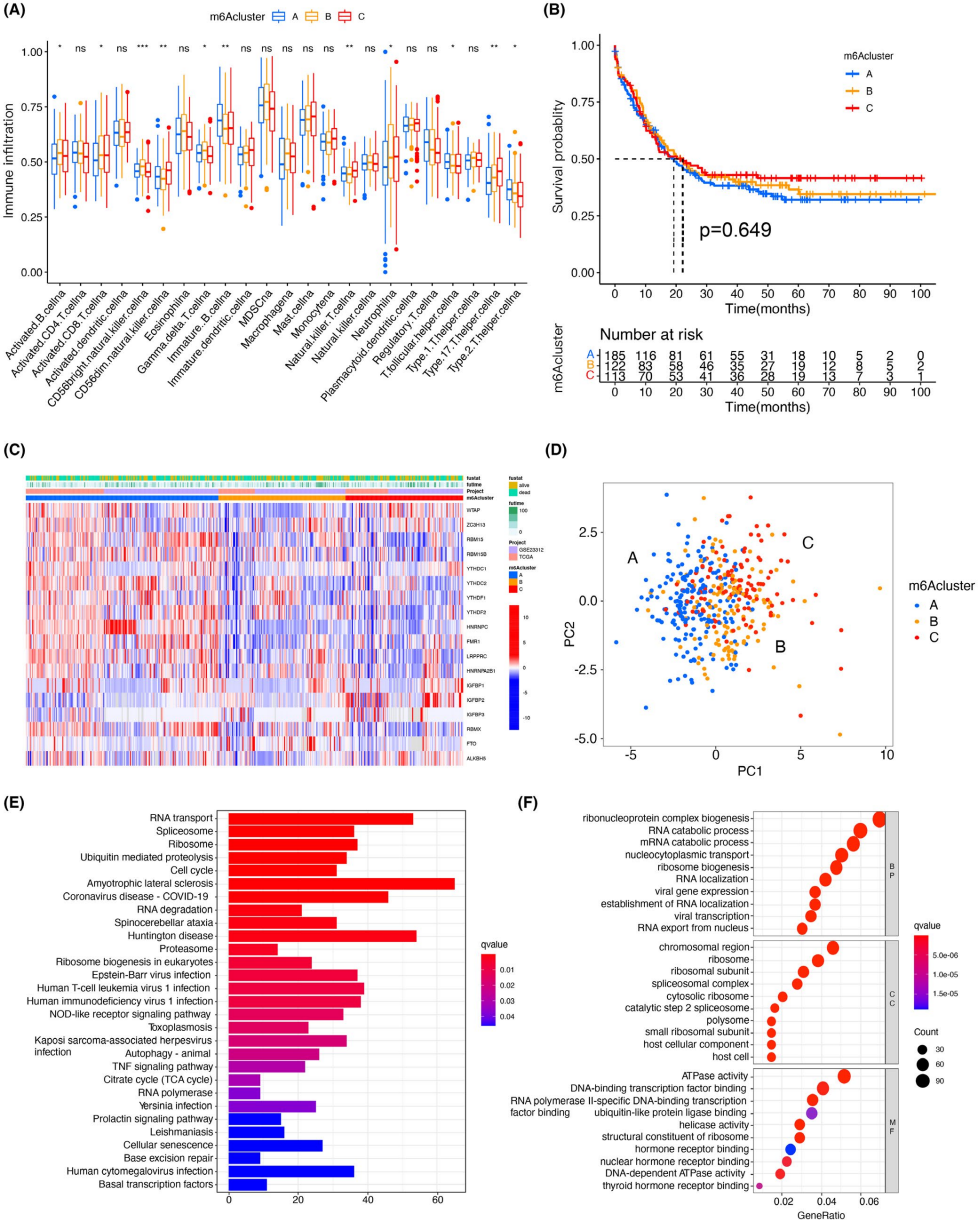
Characterization of TME cell transcriptome and infiltration features for different m6A modification patterns. (A) Abundance of TME‐infiltrating cells in the three m6A modification patterns. The lower and upper ends of the boxes indicate the interquartile range of values. Black dots in the boxes indicate outliers and lines represent medians. Asterisks represent statistical *p*‐values (*p < 0.05; **p < 0.01; ***p < 0.001). (B) Survival analysis of three m6A modification patterns, including 185 m6Acluster‐A, 122 m6Acluster‐B, and 113 m6Acluster‐C, based on survival data from 420 AML patients in the GEO and TCGA cohorts (GSE23312). (C) Survival analysis of 18 m6A in the GEO and TCGA cohorts. Regulators for unsupervised clustering. Survival status, m6Acluster, age, and item origin are used as patient annotations. Blue represents low expression and red represents high expression. (D) Principal component analysis of the transcriptome profiles of the three m6A modification patterns showed significant variation between the transcriptomes of the different modification patterns. (E) Functional annotation of m6A‐related genes using KEGG enrichment analysis. The size and distance of the gas bubbles represent the number of genes contained in the pathway and the q value, respectively. (F) Functional annotation of m6A‐associated genes using GO enrichment analysis. The color depth of the bar graph indicates the number of enriched genes

To explore the differences between these m6A modifications, we performed GSVA analysis. Gene sent enrichment score showed that, in Figure 1E, m6Acluster‐A was clearly enriched in oncogenic activation pathways, such as leukocyte trans‐endothelial migration and WNT signaling pathways; m6Acluster‐B showed cellular signaling pathways associated with full immune action, including cytokine‐cell receptor interactions, activation of chemokine pathways, etc (Figure 1E). In addition, m6Acluster‐C correlated with the NK cell‐mediated cytotoxicity (Figure 1F). Subsequent analysis of TME infiltration revealed that m6Acluster‐B was significantly enriched for innate immune infiltration, such as macrophages, natural killer cells, mast cells, eosinophils, plasma cells, and MDSC (Figure 2A). However, there was no statistical difference in the overall survival of patients corresponding to the different m6A modification status (Figure 2B). We subsequently identified three completely different m6A modification patterns by unsupervised clustering (Figure 2C,D). Differences in m6A transcriptional profiles existed among these three m6A modification patterns (Figure 2D). m6Acluster‐A was featured by elevated expression of YTHDF2, YTHDF1, RMB15, RMB15B, HNRNPC, and FMR1 as well as decreased expression of other regulators; m6Acluster‐B displayed increased level of FTO, whereas m6Acluster‐C showed IGFBP2, ZC3H13, and WTAP expression was significantly increased. The levels of genes were related to the survival of patients. We also identified seven DEGs (YTHDF2, YTHDF1, RMB15, ZC3H13, IGFBP2, HNRNPC, and WTAP) associated with the m6A phenotype using the “limma” package in R software (Figure S3). KEGG and GO analysis of the DEGs were conducted and summarize the biological procedure. These genes displayed an enrichment of biological procedure significantly associated with m6A modification, which confirms that m6A modification plays an important role in regulation of the neoplasms microenvironment (Figure 2E,F).

### 3.4 Features of the transcriptome and clinical profile of m6A‐related phenotype

**FIGURE 3:**
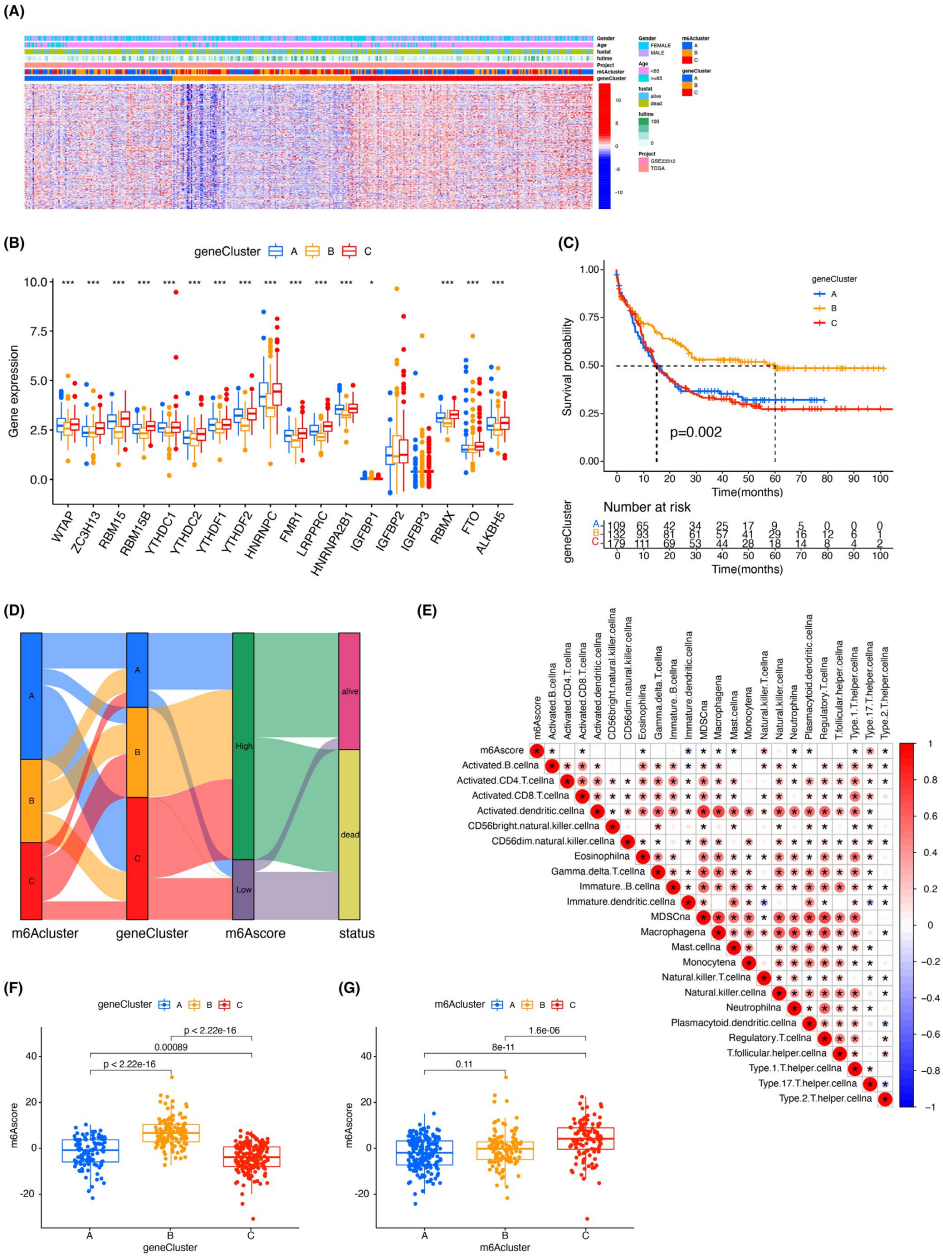
Structure of m6A signatures. (A) Unsupervised clustering of m6A phenotype‐associated genes in a total of 469 overlapping patients in the GEO and TCGA cohorts to classify patients into different genomic subtypes, referred to as m6A gene clusters A–C, respectively. (B) Expression of 18 m6A regulators in the three gene clusters. The lower and upper ends of the boxes represent the interquartile range of values. Black dots in the boxes indicate outliers and lines represent medians. Asterisks denote statistical *p*‐values (*p < 0.05; **p < 0.01; ***p < 0.001). One‐way ANOVA test was applied to detect statistical variation among the three gene clusters. (C) Kaplan–Meier curves showed that the m6A‐modified genomic phenotype was related to overall survival in 420 patients in the TCGA + GEO cohort, 109 of whom belonged to genogroup A, 132 to genogroup B, and 179 to genogroup C (*p* < 0.01, log‐rank test). (D) Alluvial plots are showing changes in m6Aclusters, geneClusters, m6Ascore, and survival states. (E) Correlations between m6Ascore and known genetic traits in the TCGA + GEO cohort using Spearman analysis. Negative correlations are marked in blue and positive correlations are marked in red. (F) Differences in m6Ascore between the three gene clusters in the TCGA + GEO cohort. Kruskal–Wallis test was applied to compare the statistical variations between the three gene clusters (*p* < 0.001). (G) Variations in m6Ascore between the three m6A modification patterns in the TCGA + GEO cohort (*p* < 0.001)

Then, we conducted an unsupervised clustering analysis based on the derived genes associated with the m6A phenotype to validate this mechanism of regulation. We also discovered three different m6A‐modified genomic types, and we designated those three clusters as m6A gene clusters A–C (Figure 3A). This suggests that three m6A methylation modification patterns were effective in AML. We discovered that neoplasm in m6A gene clusters A and C patterns exhibited poor differentiation, with high expression of both incorporated genes. The opposite way was observed in m6A gene cluster B. Older age (Median [IQR]: 54[41–62] vs. 40[34–51]) and higher mortality (63.3% vs. 51.2%) was observed in patients with m6A gene clusters A and C patterns (Figure 3A). Our data show that these three distinct gene clusters are described by other trait genes (Figure 3A). Of the 420 AML patients, 132 was for cluster B, which was proven to be related to better outcome.

In contrast, clusters A and C (109 and 179 cases) had a worse prognosis (Figure 3C). The m6A‐ modified gene also differed in the different gene sets formed by RNA‐seq clustering analysis. This is in line with the expectation of the m6A methylation modification pattern (Figure 3B). Compared to the clustering of m6A, the gene clustering is more indicative in terms of predictive value. Patients with low m6A scores have a higher mortality. Cluster A and C correspond to lower m6A scores and have poorer prognosis (Figure 3D). To better characterize the m6A traits, the correlation between available features and m6Ascore was identified (Figure 3E). Kruskal–Wallis test showed remarkable differences in m6Ascore between different groups. Cluster C displayed the minimum mediocre score.

In contrast, gene group B scored the highest median points, suggesting that poor m6Ascore may be closely associated with immune activation‐related traits. In contrast, an increased m6Ascore may be related to stromal activation‐related features (Figure 3F). More importantly, m6A cluster C had a significantly increased m6Ascore, and m6A clusters A as well as B had relatively lower median scores than other clusters (Figure 3G).

**FIGURE 4:**
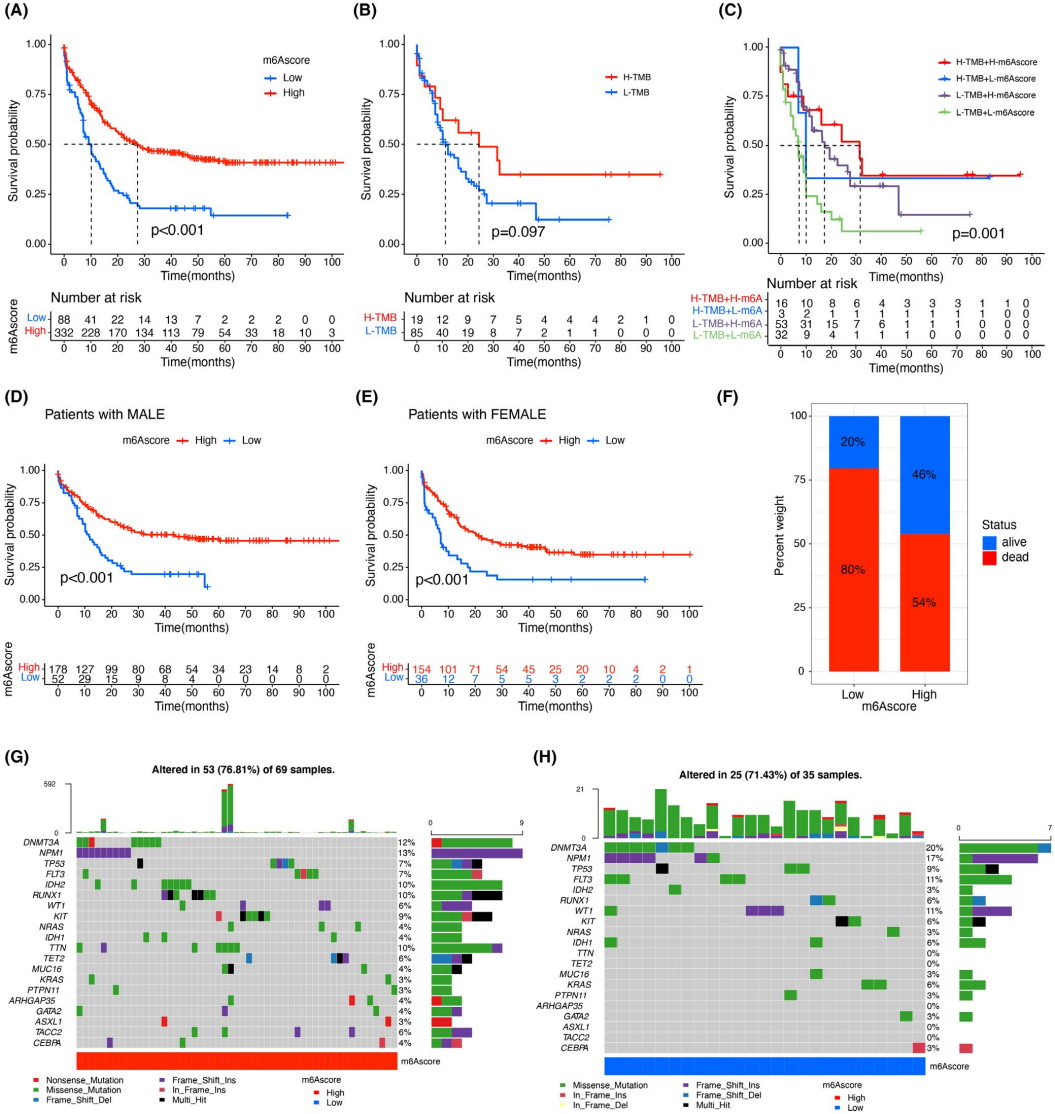
Characterization of m6A modifications and tumor mutations in TCGA + GEO molecular isoforms. (A) Survival analysis was performed using Kaplan–Meier curves for the low (88) and high (332) m6Ascore patient groups in the TCGA + GEO cohort (*p* < 0.001, log‐rank test). (B) Kaplan–Meier curves for subgroups of patients stratified by TBM (tumor mutation burden) (*p* < 0.097, log‐rank test). (C) OS analysis was conducted via Kaplan–Meier curves for subgroups of patients stratified by m6Ascore and TBM. h, high; l, low (*p* < 0.001, log‐rank test). (D) Kaplan–Meier curves for subgroups of male patients stratified by m6Ascore (*p* < 0.001, log‐rank test). (E) Kaplan–Meier curves for subgroups of female patients stratified by m6Ascore (*p* < 0.001, log‐rank test). (F) A graph of the proportion of patients alive and dead under different m6Ascore groupings. (G and H) Waterfall plot of neoplasms somatic mutations set up by those with increased m6Ascore (G) and decreased m6Ascore (H). Each column represents an individual patient. The top bar graph shows the TMB, and the numbers on the right indicate the mutation frequency of each gene. The right bar graph displays the proportion of each mutation type

Then, we determine the points of the m6Ascore in evaluating patient prognosis. The critical points specified with the “survminer” package was −6.26, and patients were classified into different m6Ascore arms. Increased m6Ascore showed a significant prognostic advantage (Figure 4A,F), with 5‐year OS twofold as high as those with an increased m6Ascore (14.4% vs. 40.9%). Multivariate analysis, including patient age and gender, confirmed that m6Ascore is an independent and reliable biomarker for patient prognosis evaluation. In particular, we investigated the survival of patients at different TMB levels. We found that the prognosis was worse in decreased TMB cluster (Figure 4B). Moreover, we grouped both m6Ascore and TMB and found that patients with low TMB levels and low m6Ascore had the worst prognosis (Figure 4C). The effect of m6Ascore on survival after differentiating subgroups by gender was consistent with previous findings (Figure 4D,E). A Spearson correlation analysis was displayed statistically between patients’ TMB and m6AScore (r = 0.91, *p*‐value = 0.016).

### 3.5 Characterization of m6A modifications in tumor somatic cell mutations and TCGA subtypes

We next identified the variation in the somatic mutation distribution between different m6Ascore in the TCGA‐LAML set via the “maftools” package. In Figure 4G,H, the high‐m6Ascore group displayed a high neoplasm mutation burden than the low‐m6Ascore set. Preclinical studies and clinical trials have shown that patients with elevated somatic TMB are associated with good prognosis. For specific genes in TCGA‐LAML, such as DNMT3A and FLT3, the mutant m6Ascore was significantly lower than the wild‐type, whereas the wild‐type and mutant m6Ascore for NPM1 and TP53 were not completely different. These results will provide new perspectives to invest the mechanism of m6A methylation modification in tumor somatic mutation, the role in immune checkpoint blockade therapy and the TME landing.

**FIGURE 5:**
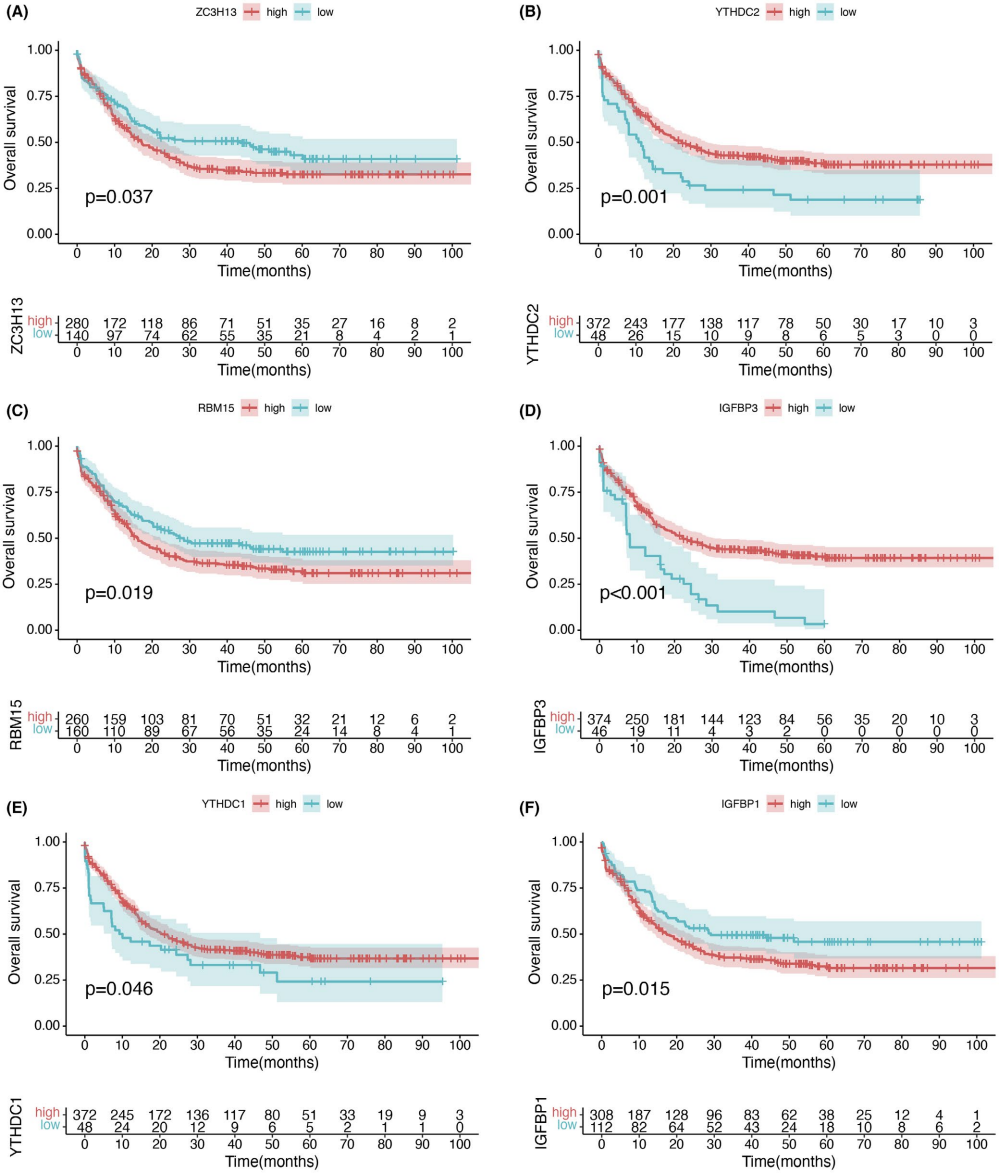
Survival curves of m6A‐related genes at different expression levels. (A) OS analysis was conducted via Kaplan–Meier curves for subgroups of patients stratified by ZC3H13 level (*p* < 0.05, log‐rank test). (B–F) Kaplan–Meier curves for subgroups of patients stratified by YTHDC2 (B), RMB15 (C), IGFBP3 (D), YTHDC1 (E), IGFBP1 (F) expression (*p* < 0.05, log‐rank test)

**FIGURE 6:**
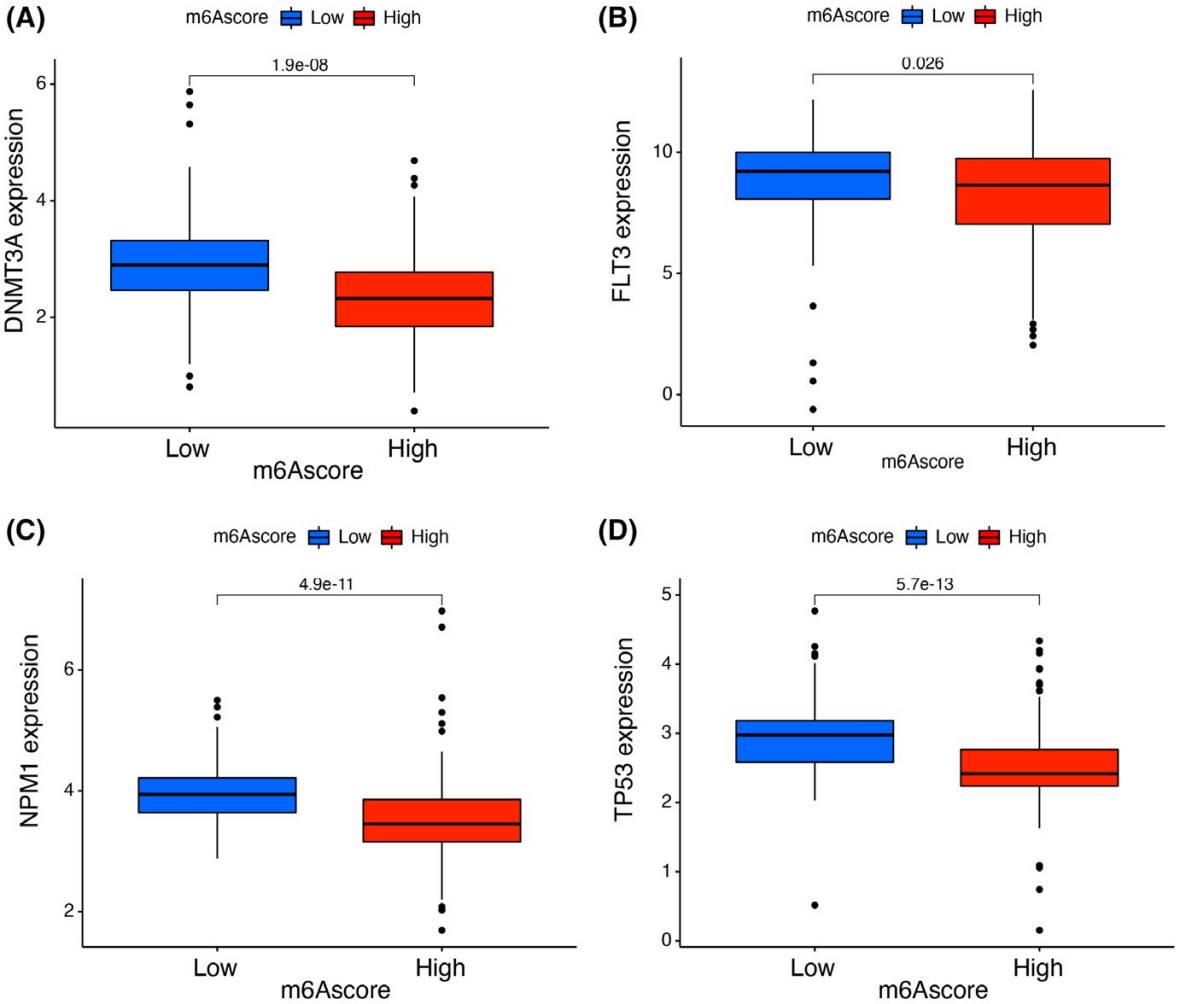
Expression of genes with high‐mutation rates under different m6Ascore. (A) Expression of DMNT3A under high m6Ascore and low m6Ascore. (B–D) FLT3 (B), NPM1 (C), TP53 (D) expression under high and low m6Ascore

Survival analysis of m6A‐related regulatory genes showed that ZC3H13 (Figure 5A), RBM15 (Figure 5C), and IGFBP1 (Figure 5D) could achieve a better prognosis with low expression. In contrast, YTHDC2 (Figure 5B), IGFBP3 (Figure 5F), YTHDC1 (Figure 5E) could obtain a better prognosis with high expression, and the differences were statistically significant. We grouped m6Ascore and discovered that the differences of the levels of AML‐related genes DNMT3A (Figure 6A), FLT3 (Figure 6B), NPM1 (Figure 6C), and TP53 (Figure 6D) at their distinct integration levels were statistically significant.

## 4 DISCUSSION

Previous evidence suggests that m6A regulatory‐related genes regulate m6A modifications in the organism and play an essential roles in antitumor effect and inflammation. Because early studies on leukemia‐m6a association were scarce and these studies focused on a single TME cell type. Determining the function of different m6A modification patterns in TME infiltration will help to guide more efficacious immunotherapeutic approaches.

There are three distinct patterns of m6A methylation modification based on 18 m6A regulators, which we reveal here. These three patterns are characterized by distinctly different TME cell infiltration. Group A is featured by adaptive immunity, responding to an immunoinflammatory phenotype; group B is featured by innate immunity and stroma, linked to an immune rejection phenotype; group C is featured by suppression of immunity, linked to an immunodeficiency phenotype. The immune rejection as well as immunodeficiency phenotypes can be considered as noninflammatory neoplasms. The immunoinflammatory phenotype, referred to as a pyogenic tumor, is characterized by a significant infiltration of immune cells in the TME.^27^, ^28^ The immunodeficient phenotype is associated with immune tolerance and lacks activated T cells.^29^ In line with the above definition, we identified cluster C as exhibiting a distinct stromal activation state confirmed the reliability of our immunophenotypic classification of the different m6A modification patterns. Thus, after fully exploring the features of TME cell infiltration induced by different m6A modification patterns.

New evidence suggests that N6‐methyladenosine (m6A) modifications of mRNA are the most abundant internal mRNA modifications^30^ involved in the differentiation, pathogenesis, and hematopoietic specification of AML.^31^ M6a modifications are conducted by the methyltransferase complex composed of the METTL14 and METTL3 isomerase cores, as well as their regulator WTAP^32^ are reversed by m6A demethylases (FTO^33^ and AlkBH5^34^), known as m6A erasers. Previous investigations have uncovered the requirement of METTL14, METTL3, and FTO in leukemic transformation.^31^, ^35^ However, while m6A modifications regulate mRNA production, translation, and degradation,^10^ the role of m6A on leukemic transformation has not been investigated.

We built on these studies by analyzing 18 m6A‐related genes and overall scoring to analyze m6A modifications in AML patients as a whole. We identified three distinct groups of m6A modifications. These three TME cell infiltration patterns are characterized by a high degree of concordance with immune rejection, immune inflammatory, and immune inert phenotypes. It is demonstrated that assessment of m6A modification patterns can predict the stage of neoplasms inflammation, genetic variation, patient prognosis, and subtype. The low m6Ascore is marked by increased immune activation and mutational burden, indicating an inflammatory phenotype of TME with a 5‐year survival rate of 14.4%. Lack of adequate immune infiltration as well as stromal activation was observed in the high‐m6Ascore subtype, indicating an immune rejection and noninflammatory phenotype of TME with a 5‐year survival rate of 40.9%. Data from both cohorts confirm that patients with a higher m6Ascore show a clear therapeutic advantage and clinical benefit but external validation is needed in the future.

In conclusion, m6A modifications play a substantial role in the process of TME diversity and complexity. Assessing m6A modification patterns in AML patients will contribute to improving our knowledge of TME infiltration features and lead to more effective immunotherapeutic treatment strategies.

## CONSENT FOR PUBLICATION

We have got consent from all the authors for publication.

## ETHICAL APPROVAL AND CONSENT TO PARTICIPATE

The Clinical Research Ethics Committee approved clinical data in First Affiliated Hospital of Soochow University.

## CONFLICT OF INTEREST

The authors declare that there is no competing interest.

## AUTHOR CONTRIBUTION

HSY and QJQ conducted the analysis and drafted the manuscript. FK completed research studies and analyzed data. WH contributed to the data analysis and manuscript writing. TYQ adds to the collection and analysis of clinical data. HY and WDP contributed to the research design, data analysis, writing the manuscript, and supervision of the study. All the authors approved the final manuscript.

## Supporting information

Fig S1

Fig S2

Fig S3

## Data Availability

All the data and materials were available if necessary.
