# Supplementary figures and images for: Characterization of m6A regulator‐mediated methylation modification patterns and tumor microenvironment infiltration in acute myeloid leukemia
Source: Cancer Med. 2022 Jan 13;11(5):1413–26. doi: 10.1002/cam4.4531 (PMC8894699; doi:10.1002/cam4.4531)

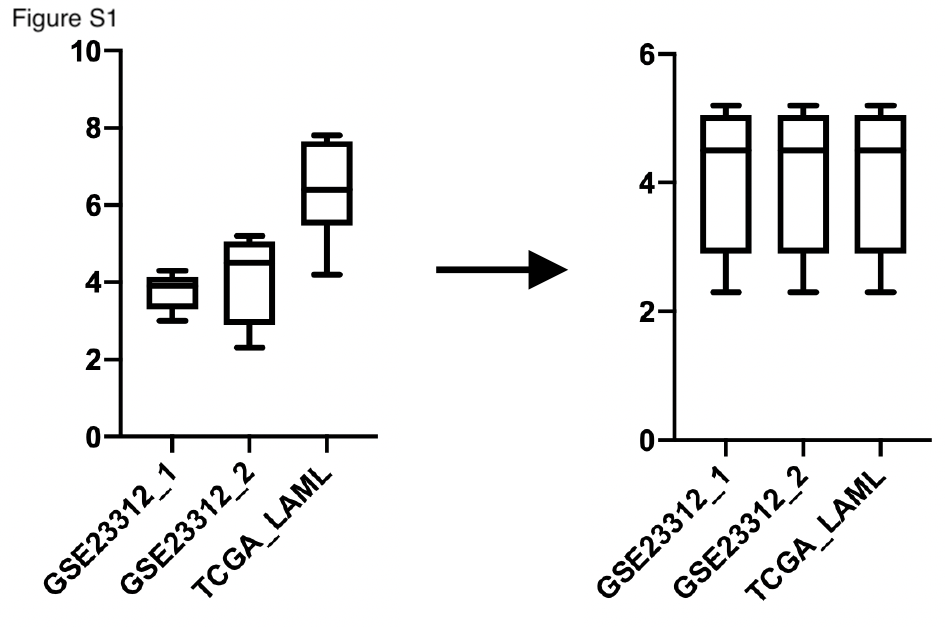

Supplement: Supplementary file 1 — Fig S1 [file CAM4-11-1413-s002.tif]

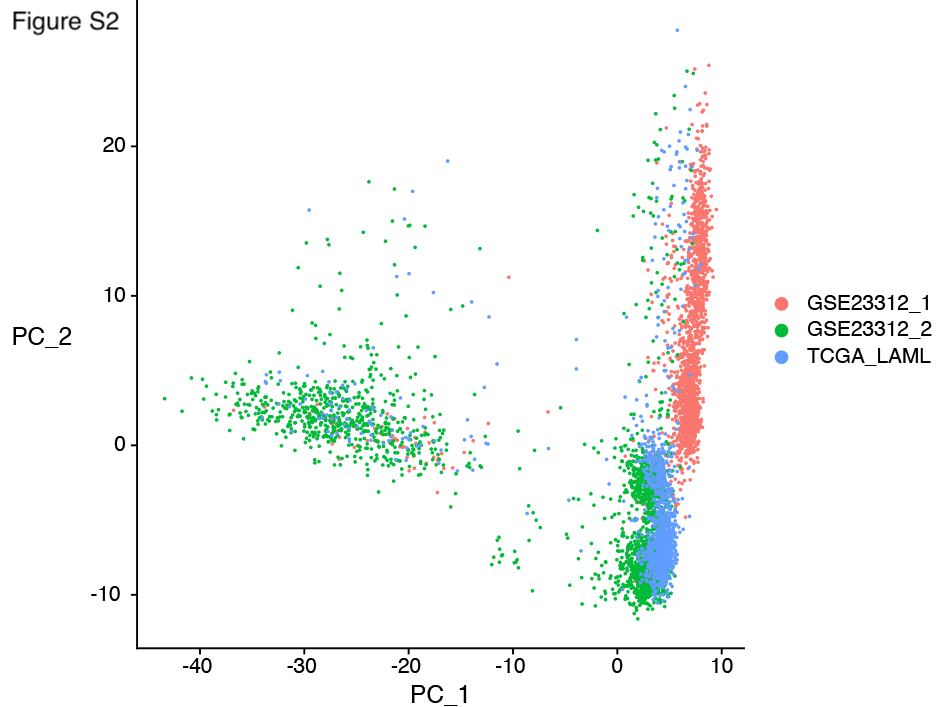

Supplement: Supplementary file 2 — Fig S2 [file CAM4-11-1413-s003.tif]

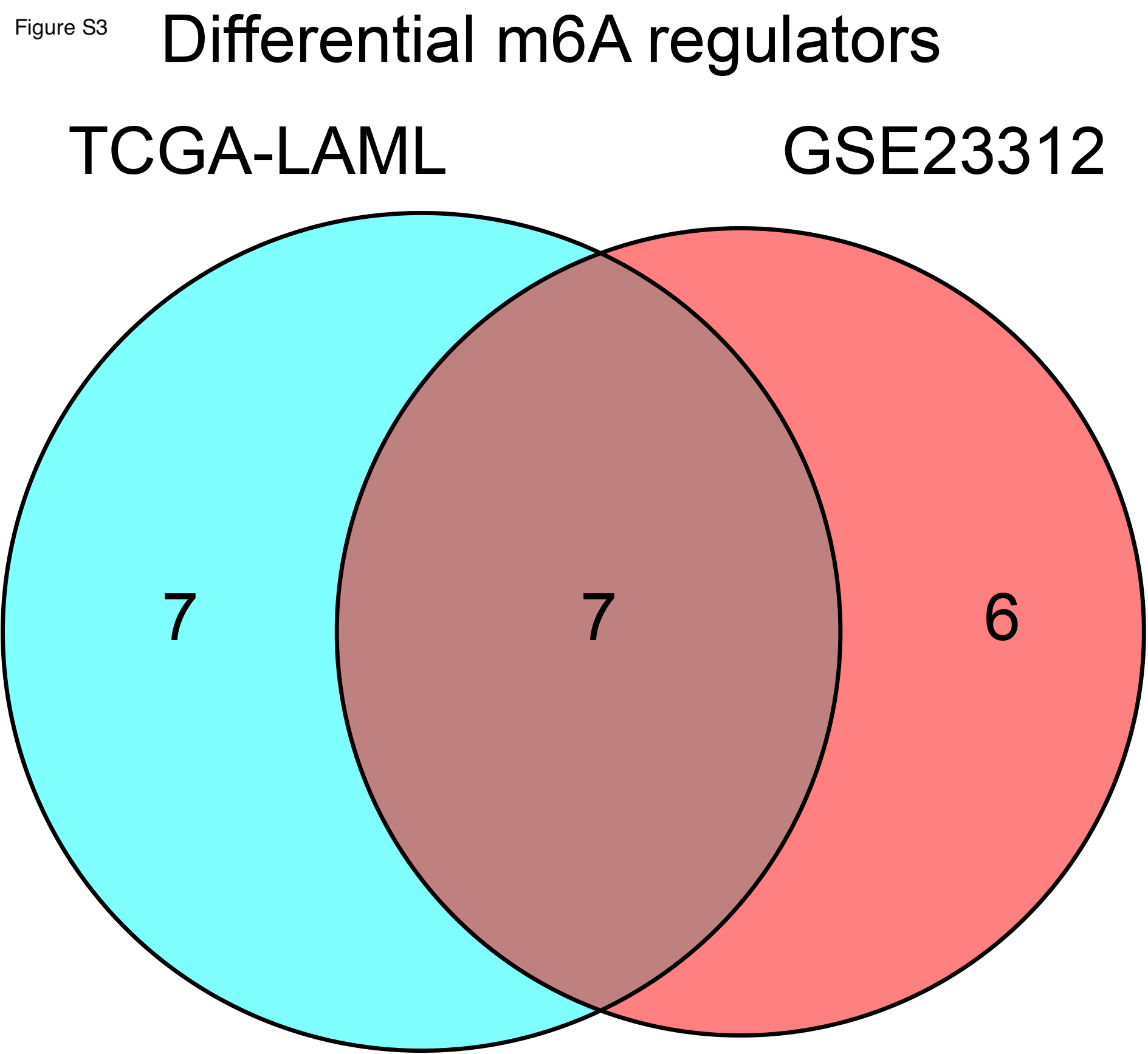

Supplement: Supplementary file 3 — Fig S3 [file CAM4-11-1413-s001.tif]
